# Investigating links between co-rumination and personality, social functioning, and emotional well-being in a representative sample of adults

**DOI:** 10.1371/journal.pone.0349391

**Published:** 2026-05-14

**Authors:** Thomas F. Denson, Michelle L. Moulds, Jessica R. Grisham

**Affiliations:** School of Psychology, University of New South Wales, Sydney, New South Wales, Australia; Manchester Metropolitan University, UNITED KINGDOM OF GREAT BRITAIN AND NORTHERN IRELAND

## Abstract

Co-rumination occurs when people discuss problems in-depth, engage in speculation about their problems, and focus on negative affect. Current evidence suggests that co-rumination involves a paradox: whilst it can strengthen relationship bonds, co-rumination can also adversely impact mood (e.g., lead to anxiety). Most of the research on this topic to date has been conducted with children and adolescents. In this survey study, we examined the correlates of co-rumination in a representative sample of 495 adult residents of the UK (aged 18–87, stratified by age, gender, and ethnicity). In regression models, well-validated self-report measures of individual differences in perspective-taking, extraversion, agreeableness, anxiety, and measures of well-being were associated with self-reported co-rumination. Structural equation modeling showed that co-rumination mediated the relationship between some of the individual difference factors and both reduced social distress (i.e., lower loneliness and improved relationships well-being) and increased anxiety. Effect sizes were typically small. In most models, age was inversely associated with co-rumination. The emerging picture, at least for adults, is that whilst co-rumination may be associated with relationship satisfaction and well-being more broadly, it is also associated with higher levels of anxiety.

## Introduction

Rumination is perseverative thinking about problems, events in the past, and current circumstances [1]. The nature and consequences of rumination have been studied extensively, and the adverse consequences of this cognitive process are well-established, particularly in clinical disorders (e.g., [2]). As an extension of this individual, intrapersonal process, *co-rumination* refers to ruminating with others (i.e., repetitively discussing and re-hashing problems and analyzing their causes and consequences) [3–7]. Co-rumination commonly occurs in the context of close interpersonal relationships, such as with friends or romantic partners. There is evidence that co-rumination is paradoxically linked to both beneficial social (e.g., closer relationships) and negative emotional outcomes (e.g., internalizing symptoms) [5,8]. There is reasonable consistency in these findings across studies, and the findings consistently suggest that co-rumination is perceived as socially beneficial, but it can also increase negative emotions.

Research in this area has predominantly examined co-rumination in the context of friendships in children and adolescents (e.g., [9–11]). Adolescent females report more co-rumination than their male counterparts [5]. Spendelow et al.’s [12] meta-analysis confirmed high levels of co-rumination in females across child, adolescent and young adult samples. Adolescence is no doubt an important and relevant developmental period in which to study co-rumination – as friendship dyads and groups of teenagers collectively grapple with identity formation and relationships [13]. However, it is important that we broaden the focus and investigate co-rumination beyond this developmental window in order to inform scientific understanding of the prevalence and correlates of co-rumination in adulthood. To date, far fewer studies have investigated co-rumination outside adolescent relationships (e.g., [14]). In adulthood, individuals’ sense of identity may be more resistant to change; thus, co-rumination in adults may be less prevalent or angst-driven as that observed in adolescents. However, if adults do co-ruminate – and if their co-rumination yields different costs and benefits than it does for adolescents – it follows that interventions to reduce co-rumination should be tailored to each group.

The literature’s current focus on younger populations overlooks the possibility that co-rumination occurs in other stages of life (e.g., adulthood, older age) and relationship contexts in which peer relationships are highly salient (e.g., workplaces), as well as within groups of individuals with mutual interests or concerns (e.g., new parents’ groups, climate activist groups). Given the range of contexts in which co-rumination can occur, it is important that research examines whether the links between co-rumination and both beneficial and adverse outcomes observed in young people are also evident in other contexts and developmental periods.

### Conceptual rationale

Acknowledging these gaps in the literature, we conducted a cross-sectional survey study to characterize the prevalence and individual difference correlates of co-rumination in adulthood (i.e., in a representative sample of adults in the UK), and determine whether the relationships between individual difference variables and the tendency to engage in co-rumination mirror those reported for individual rumination. Our theoretical approach involves a conceptual rationale of co-rumination that is associated with demographics, social cognitive variables, relationship factors, the Big 5 personality dimensions, individual rumination, and well-being. We chose these groups of variables for their potential relevance to co-rumination, which we describe below. In addition to our focus on adulthood, by documenting relationships between theoretically relevant clusters of variables and co-rumination, this research advances prior theorizing on co-rumination. First, we examined the association between co-rumination and both age and gender, to determine whether co-rumination was more likely in younger individuals and females. Second, we were also interested in whether individuals who demonstrate higher levels of social cognition (as measured by empathic concern, emotional intelligence and perspective-taking) report higher levels of co-rumination. In the case of perspective-taking, given evidence that individual rumination is negatively correlated with perspective-taking [15] and may negatively impact upon social interactions [16], we examined whether co-rumination was associated with impaired social cognition. However, we considered the converse also possible: that as a social and interpersonal process, co-rumination may well be linked to enhanced (rather than diminished) perspective-taking, as is the case in early adolescence [17]. Indeed, partner responsiveness has been identified as an important process during co-rumination in young adults [7].

Third, we investigated potential associations between co-rumination and relationship factors. In particular, we examined whether co-rumination would be associated with feelings of well-being in relationships and reduced loneliness. As prior work suggests that co-rumination may improve relationship closeness, we considered the possibility that more co-rumination would be related to improved feelings of well-being in relationships and a reduced sense of loneliness.

Fourth, the five-factor model of personality informed our approach to examining the relationships between the Big 5 and co-rumination. Previous studies examining associations between the Big Five personality traits and the tendency to engage in (individual) rumination have demonstrated that rumination is correlated with higher levels of neuroticism [18] and lower levels of conscientiousness [19], extraversion [20], and agreeableness [18]. However, links between personality traits and the more interpersonally-driven construct of co-rumination have not yet been explored in the context of a general community sample. Because co-rumination is a social behavior, we expected that the prosocial orientation associated with agreeableness and the gregarious nature of people high in extraversion would be associated with increased co-rumination. We also included a measure of individual differences in rejection sensitivity. We reasoned that this trait could conceivably be higher or lower in individuals who habitually co-ruminate. That is, people who tend to co-ruminate may feel secure in their relationships and hence lower in rejection sensitivity or, alternatively, co-ruminators may co-ruminate because they are sensitive to rejection and try to process perceived rejections with a close other.

Fifth, on the basis of established links between individual rumination and psychopathology [1], as well as co-rumination and internalizing symptoms [5], we tested whether people who tend to ruminate at the individual level are also likely to ruminate with others. While recent findings suggest a relationship between co-rumination and intrapersonal perseverative cognition [14], the link between co-rumination and the tendency to engage in individual rumination warrants investigation. We therefore explored the association between co-rumination and the trait tendency to engage in individual rumination about specific emotional content (i.e., depression and anger). In addition, the literature on individual rumination has demonstrated that rumination has differential outcomes depending on whether it is abstract/analytical (focusing on causes, meanings, implications: e.g., “*Why did this happen? Why do I feel like this?”*) or concrete (focusing on specific details: e.g., “*What happened? How do I feel right now?”*) – with the former leading to a range of adverse consequences [2,21]. Despite the well-established differences in these two styles of rumination, no study to date has examined the way in which people discuss problems during co-rumination; and in particular, whether it is characterized by a more abstract (rather than concrete) style. Drawing on evidence that individual rumination is typically abstract (rather than concrete), we also examined whether participants reported that their co-rumination was more abstract in nature.

Finally, we also examined the interrelationship of co-rumination and indices of overall functioning, including psychological (e.g., depression, anxiety, stress symptoms), physical, and interpersonal well-being (e.g., relationship well-being and loneliness). Based on prior research, we expected a positive relationship between co-rumination and relationships well-being; however, we were uncertain as to whether the social well-being benefits would extend to the physical well-being domain or mental health. Because the literature generally shows relationships between co-rumination and anxiety, we expected to observe this relationship in our sample as well.

### The present research

We administered a survey to a representative sample of UK adults. Participants completed measures of demographics, social cognitive variables, relationships factors, the Big 5 personality dimensions, individual rumination, and well-being. We then analyzed the relationships between these variables and co-rumination using regression models. Based on the results of the regression analysis, we computed a structural equation model in which co-rumination was postulated to statistically mediate the effects of the individual difference measures on positive (e.g., social well-being) and negative outcomes (e.g., anxiety).

## Method

### Ethics statement and power analyses

The research was approved by the University of New South Wales’ Human Research Ethics Committee (Project ID#8736). The research was not pre-registered. Data were collected on May 7 and 8, 2025. All participants provided informed consent electronically via mouse click. A power analysis with the *pwr* package in R [22] suggested that 346 participants would be required to detect a correlation of .15 at 80% power, α_two-tailed_ = .05. Similarly, a power analysis for the multiple regression analyses indicated that for power at 80%, data from 309 participants would suffice to detect a small-to-medium effect, *f*^*2*^ = .05, with eight predictors, α = .05_two-tailed_. We used the *WebPower* package [23]. We also conducted a power analysis for the structural equation model using the *semPower* package [24], which revealed that data from 434 participants would suffice to detect an RMSEA at .05, with 80% power, α = .05_two-tailed_.

### Participants

Participants were a representative sample of 500 Prolific users in the UK. Three participants were excluded for failing an attention check, resulting in a sample of 497 (251 women, 244 men, 1 non-binary, and 1 ‘other’). The data from these latter two participants were excluded from analyses, leaving a final sample of 495 (*M*_age_ = 46.65, *SD*_age_ = 15.44, ranging from 18 to 87 years old). Participants were primarily White (85%), followed by South Asian (5%), African (3%), other ethnicities (3%), East or Southeast Asian (2%), mixed ethnicities (1%), and Middle Eastern (<1%). On average, participants reported co-ruminating a moderate amount (*M* = 3.70, *SD* = 1.45) on a 7-point scale from 1 = not at all to 7 = every day).

### Materials

**Co-rumination questionnaire.** Participants completed a modified version of the co-rumination questionnaire [3], which measures the extent to which people co-ruminate. The original measure was designed to assess co-rumination among child and adolescent friendship dyads (i.e., ‘with your best or closest friends’). We modified this 27-item measure to broaden the focus and make it applicable to an adult sample with the following instructions: “For the next set of questions, please think about a person in your life who is close to you (e.g., a friend, romantic partner, family member, co-worker) and with whom you tend to discuss problems. We shall refer to this person as your confidant (that is, a person you confide in). Think about this person as you answer the following questions.” We also modified the individual items to reflect these instructions; e.g., “We spend most of our time together talking about problems that my confidant or I have” (1 = Not at all true; 5 = Really true). The original scale has good psychometric properties [25] and our modified scale showed excellent internal consistency reliability in the current sample (α = .97). Because we modified the scale, we conducted a confirmatory factor analysis to further examine construct validity. Although fit indices were mixed, we generally replicated the three-factor structure (i.e., rehashing, mulling, encouraging problem talk) observed by Davidson et al. [25], χ^2^(296) = 977.58, χ^2^/df ratio = 3.30, CFI = .91, RMSEA = .078, SRMR = .050. However, we used a single factor measure for parsimony, because we did not have subscale-specific predictions, it is the most common way to use the scale [26], and because the factors were highly inter-correlated, *r*s > .80.

**Social-cognitive traits.** Participants completed the empathic concern and perspective-taking subscales of the Interpersonal Reactivity Index [27]. Items included “I am often quite touched by things that I see happen” and “I believe that there are two sides to every question and try to look at them both”, for empathic concern and perspective-taking, respectively (1 = Does not describe me well; 5 = Describes me well). Due to an error, the perspective-taking subscale consisted of 5 rather than 7 items. Participants also completed the 30-item short form of the Trait Emotional Intelligence Questionnaire [28]. Items assessed numerous aspects of emotional intelligence (e.g., “I can deal effectively with people” and “I often pause and think about my feelings”) (1 = Completely Disagree; 7 = Completely Agree).

**Relationships.** Participants completed measures of loneliness and well-being within relationships. The UCLA Loneliness Scale consists of 20-items assessing the degree of loneliness currently experienced by respondents (e.g., “How often do you feel part of a group of friends?”) (1 = never, 4 = always) [29]. To assess relationships well-being participants completed the Relationships Well-being subscale of the BBC Well-being Scale [30]. This subscale assesses satisfaction and well-being with social relationships (e.g., “Are you happy with your friendships and personal relationships?) (1 = Not at all; 5 = Extremely).

**Personality.** Participants completed the Ten-item Personality Inventory [31], which is a brief measure of the Big Five personality traits. Participants respond to pairs of adjectives (two pairs per dimension) and rate the extent to which the pair of adjectives apply to them (1 = Disagree strongly; 7 = Agree strongly). Scores are computed by averaging both pairs of adjectives for extraversion (e.g., “extraverted, enthusiastic”), agreeableness (e.g., “sympathetic, warm”), conscientiousness (e.g., “dependable, self-disciplined”), neuroticism (e.g., “anxious, easily upset”), and openness (e.g., “open to new experiences, complex”).

To assess sensitivity to interpersonal rejection, participants completed the Adult Rejection Sensitivity Questionnaire [32]. The scale consists of 9 situations that might elicit fear of rejection (e.g., “You call a friend when there is something on your mind that you feel you really need to talk about”). Participants then respond to two items for each situation to indicate how concerned they would be about being rejected and how much they expect that rejection would be likely (1 = Very unconcerned to 6 = Very concerned and 1 = Very unlikely to 6 = Very likely). The concern items are multiplied by the expectation items and averaged across the 9 situations.

**Rumination.** To assess emotion specific rumination styles, we included measures of depressive rumination and angry rumination at the individual level. For depressive rumination, participants completed the 5-item Brooding subscale of the Ruminative Response Scale in which participants are asked about how they think when they are depressed, sad, or down (e.g., “Why do I have problems other people do not have?”) (1 = Almost never; 4 = Almost always) [33].

To assess angry rumination, participants completed the 10-item Angry Rumination subscale of the Displaced Aggression Questionnaire [34]. This measure assesses individual differences in the tendency to ruminate on anger provocations (e.g., “I keep thinking about events that angered me for a long time”) (1 = Very uncharacteristic of me; 7 = Very characteristic of me).

Finally, participants rated the extent to which their co-rumination was abstract versus concrete in nature. Specifically, we specified: “I would like you to rate, on a 0–100 scale, how much your discussions about your problems with your confidant tend to be abstract and general (i.e., focused on general concepts or themes – e.g., why something happened) as opposed to concrete (i.e., focused on specific details or situations – e.g., exactly what happened), where 0 = ‘not at all abstract, completely concrete) and 100 = ‘extremely abstract, not at all concrete’”. The mean rating on the abstract/concrete scale was close to the midpoint, indicating that participants overall did not have an overwhelming tendency to co-ruminate in either an abstract or a concrete manner (*M* = 45.00, *SD* = 22.39).

**Well-being.** To assess psychological and physical well-being, participants completed the Psychological Well-being and Physical Well-being subscales of the BBC Well-being Scale [30]. The Psychological Well-being subscale measures satisfaction with various aspects of life (e.g., “Do you feel you have a purpose in life”) (1 = Not at all, 5 = Extremely). The Physical Well-being subscale measures satisfaction with one’s abilities to physically enjoy life (e.g., “Are you happy with your ability to perform daily living activities”).

**Mental Health.** To assess mental health, participants completed the Depression, Anxiety, and Stress Scales [35]. Seven items each assessed the extent to which participants experienced depression over the past week (e.g., “I found it difficult to work up the initiative to do things”), anxiety (e.g., “I felt I was close to panic”), and stress (e.g., “I found it hard to wind down”) (1 = None at all, 4 = Almost always).

### Statistical analyses

All data were analyzed in R [36]. We handled missing data with multiple imputations using the *mice* package [37]. Specifically, we created 100 imputed data sets using predictive mean matching. This procedure fills in missing data by computing each participant’s scores based on other data in the data set. It then runs the analysis on 100 imputed data sets and pools the results. We calculated Pearson’s correlations and conducted ordinary least squares regression on the pooled imputed data. Models were conceptualized according to various categories of predictors: social cognition, relationships, personality, rumination, and well-being. The percentage of missing data ranged from 0.00 to 5.25%. Trace plots and density plots were inspected and they were acceptable. We examined the fraction of missing information (fmi) and lambda values from the regression models as indicators of the extent to which missingness may have impacted the results. We then used the lavaan.mi package to conduct the structural equation modelling (SEM) analyses on the imputed data [38]. Because the data violated the assumption of multivariate normality, we used robust fit statistics and robust standard errors to evaluate the model. We relied on Hu and Bentler’s [39] conventions to evaluate model fit. These authors suggested that CFI values > .95, RMSEA values < .06, and SRMR values < .08 indicate good model fit.

## Results

Table 1 shows the descriptive statistics and internal consistency reliabilities. Table 2 shows the correlations among the study variables. Age was inversely correlated with co-rumination, suggesting that it is more frequent among younger people. Among the social cognition variables, co-rumination was modestly positively correlated with empathy, perspective-taking, and emotional intelligence. Among the relationship variables, co-rumination was positively correlated with relationships well-being and negatively with loneliness. Among the personality variables, extraversion and agreeableness were positively correlated with co-rumination and rejection sensitivity was negatively correlated with co-rumination. For the well-being measures, co-rumination was correlated with greater anxiety but was unrelated to depression and stress. However, co-rumination was also positively correlated with greater psychological and physical well-being.

**Table 1:** Descriptive statistics of the study measures.

	*M*	*SD*	Distribution	Cronbach’s α
Co-rumination questionnaire	3.07	0.83	1.00–5.00	.97
Empathic concern	3.87	0.78	1.00–5.00	.86
Perspective-taking	3.77	0.77	1.00–5.00	.77
Emotional Intelligence	4.64	0.96	1.53–7.00	.93
Loneliness	2.30	0.66	1.00–3.90	.95
Relationships well-being	3.26	1.00	1.00–5.00	.89
Extraversion	3.57	1.55	1.00–7.00	.72
Agreeableness	5.09	1.23	1.00–7.00	.52
Conscientiousness	5.29	1.27	1.00–7.00	.63
Neuroticism	3.48	1.50	1.00–7.00	.77
Openness	4.63	1.28	1.50–7.00	.50
Rejection sensitivity	10.51	5.02	1.00–31.44	.85
Brooding	1.98	0.69	1.00–4.00	.86
Angry rumination	4.03	1.75	1.00–7.00	.97
Psychological well-being	3.25	0.4	1.00–5.00	.95
Physical well-being	3.12	0.86	1.00–5.00	.87
Depression	1.71	0.71	1.00–4.00	.93
Anxiety	1.46	0.51	1.00–3.71	.85
Stress	1.81	.63	1.00–4.00	.89

**Table 2:** Correlations among the study variables. Bolded correlations are significant (*p* < .05).

Variable	1	2	3	4	5	6	7	8	9	10	11	12	13	14	15	16	17	18	19	20
1. Co-rumination	**--**																			
2. Age	**−.16**	--																		
3. Empathy	**.16**	.06	--																	
4. Perspective-taking	**.26**	.03	**.53**	--																
5. Emotional intelligence	**.16**	**.16**	**.26**	**.48**	--															
6. Loneliness	**−.17**	**−.10**	**−.10**	**−.29**	**−.75**	--														
7. Relationships wellbeing	**.26**	.02	**.14**	**.30**	**.72**	**−.78**	--													
8. Rejection sensitivity	**−.10**	**−.09**	−.08	**−.29**	**−.64**	**.65**	**−.62**	--												
9. Openness	**.11**	−.03	**.26**	**.32**	**.48**	**−.26**	**.30**	**−.22**	--											
10. Conscientiousness	.05	**.25**	.08	**.24**	**.54**	**−.37**	**.37**	**−.30**	**.20**	--										
11. Extraversion	**.15**	.06	**.18**	**.18**	**.50**	**−.45**	**.41**	**−.36**	**.33**	.11	--									
12. Agreeableness	**.19**	.02	**.50**	**.50**	**.43**	**−.32**	**.31**	**−.22**	**.30**	.19	**.15**	--								
13. Neuroticism	−.06	**−.20**	−.04	**−.27**	**−.72**	**.57**	**−.51**	**.49**	**−.31**	**−.43**	**−.33**	**−.36**	--							
14. Angry rumination	.07	**−.15**	−.05	**−.21**	**−.56**	.**55**	**−.50**	**.46**	**−.22**	**−.32**	**−.21**	**−.32**	**.59**	--						
15. Depressive rumination	.09	**−.26**	.03	**−.11**	**−.57**	**.59**	**−.44**	**.50**	**−.15**	**−.36**	**−.20**	**−.20**	**.58**	**.57**	--					
16. Depression	−.02	**−.18**	−.07	**−.22**	**−.70**	**.63**	**−.57**	**.49**	**−.23**	**−.43**	**−.29**	**−.26**	**.60**	**.48**	**.65**	--				
17. Anxiety	**.16**	**−.29**	.00	**−.14**	**−.48**	**.43**	**−.31**	**.39**	**−.13**	**−.32**	**−.17**	**−.16**	**.49**	**.43**	**.66**	**.62**	--			
18. Stress	.09	**−.27**	−.01	**−.19**	**−.57**	**.52**	**−.42**	**.44**	**−.19**	**−.33**	**−.19**	**−.24**	**.60**	**.56**	**.69**	**.72**	**.77**	--		
19. Psychological well-being	**.18**	.07	**.14**	**.32**	**.83**	**−.72**	**.81**	**−.56**	**.38**	**.45**	**.43**	**.29**	**−.67**	**−.52**	**−.52**	**−.69**	**−.38**	**−.51**	--	
20. Physical well-being	**.15**	.08	.07	**.25**	**.67**	**−.62**	**.71**	**−.51**	**.31**	**.47**	**.30**	**.22**	**−.55**	**−.47**	**−.42**	**−.57**	**−.34**	**−.45**	**.81**	--

Table 3 presents the results from the regression models. The very low fmi and lambda values suggest that missing data did not affect the outcomes. Furthermore, all variance inflation factors (VIFs) were < 4.00, suggesting multicollinearity was not a problem. For the social cognitive variables, perspective-taking was a significant predictor. In the relationships model, relationships well-being predicted co-rumination. In the personality model, extraversion and agreeableness were both positive predictors. For the well-being model, anxiety symptoms and psychological well-being both positively predicted co-rumination. Age remained a significant predictor in each of the models.

**Table 3:** Regression models predicting co-rumination.

	*b*	*SE*	*t-test*	*df*	*p*-value	*R* ^*2*^	*fmi*	*λ*
**Social Cognition**						.10		
Intercept	2.21	.26	8.50	470.04	<.0001		.03	.03
Age	−0.01	.002	−4.16	474.66	<.0001		.03	.02
Male Gender	−0.01	.07	−0.16	474.84	.88		.03	.02
Empathy	0.04	.06	0.77	469.52	.44		.04	.03
Perspective-taking	0.21	.06	3.48	473.73	.0005		.03	.02
Emotional intelligence	0.08	.04	1.73	468.41	.08		.04	.03
**Relationships Model**						.10		
Intercept	2.55	.42	6.12	446.53	<.0001		.07	.07
Age	−0.01	.002	−3.64	475.77	.0003		.03	.02
Male Gender	−0.07	.07	−0.95	478.30	.34		.02	.02
Loneliness	.06	.09	0.66	437.76	.51		.08	.08
Relationships wellbeing	0.25	.06	4.19	452.30	<.0001		.06	.06
**Personality Model**						.09		
Intercept	2.44	.39	6.26	453.75	<.0001		.06	.05
Age	−.01	.002	−3.86	472.30	.0001		.03	.02
Male Gender	0.003	.08	0.04	472.31	.97		.03	.02
Openness	0.004	.03	0.13	452.20	.90		.06	.05
Conscientiousness	0.03	.03	0.97	466.42	.33		.04	.03
Extraversion	0.07	.03	2.61	470.96	.009		.03	.02
Agreeableness	0.12	.03	3.53	469.12	.0005		.03	.03
Neuroticism	0.03	.03	0.79	462.16	.43		.04	.04
Rejection sensitivity	−0.01	.009	−0.69	467.05	.49		.03	.03
**Rumination Model**						.03		
Intercept	3.36	.19	17.31	475.18	<.0001		.03	.02
Age	−0.01	.003	−3.17	476.15	.002		.03	.02
Male Gender	−0.08	.08	−1.10	479.06	.27		.02	.02
Angry rumination	0.01	.03	0.49	473.20	.63		.03	.03
Depressive rumination	0.04	.07	0.57	457.73	.57		.05	.05
**Well-being Model**						.11		
Intercept	1.93	.33	5.81	458.26	<.0001			
Age	−0.01	.002	−2.43	469.47	.015		.05	.05
Male Gender	−0.11	.07	−1.47	472.99	.14		.03	.03
Depression	−0.02	.09	−0.24	465.21	.81		.03	.02
Anxiety	0.34	.12	2.93	463.38	.004		.04	.04
Stress	0.08	.10	0.77	456.10	.44		.05	.05
Psychological well-being	0.21	0.08	2.65	463.17	.008		.04	.04
Physical well-being	0.06	.07	0.85	469.87	.39		.03	.03

The next step was to test a proposed model showing (a) predictors of co-rumination, and (b) the relationships between co-rumination and psychological and physical well-being, social distress, and anxiety. As predictors of co-rumination, we used the significant individual difference variables from the regression models: perspective-taking, extraversion, agreeableness, and age. Indicators for the latent well-being variables were those that were significant in the regression models. We created latent variables to get a less biased measure of the constructs and to simultaneously model the relationships among the variables. The indicators for the social distress model were the Relationships Well-being subscale and the UCLA loneliness scale. The indicators for the well-being latent variable were the Psychological and Physical Well-being measures. We used the Anxiety subscale from the DASS-21 to model anxiety. The measurement model was a good fit to the data, χ^2^(1) = 0.14, χ^2^/df ratio = 0.14, CFI = 1.00, RMSEA = .000, SRMR = .004.

**Fig 1:**
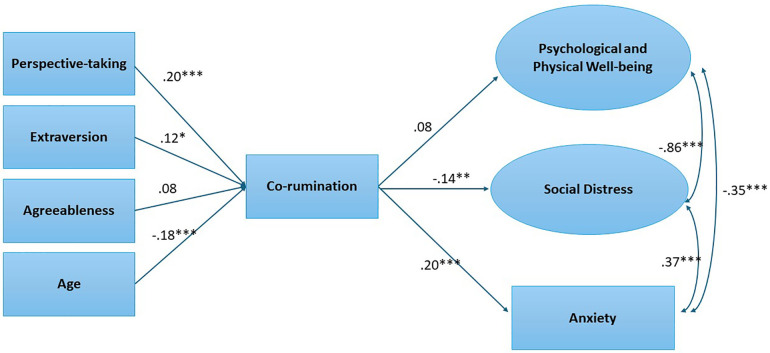
Structural equation model showing predictors of co-rumination and the relationships between co-rumination and psychological and physical well-being, social distress, and anxiety. Estimates are standardized. Not shown are the direct paths from the predictors of co-rumination to the latent variables, anxiety, or the indicators. ***p < .001, *p < .05.

We then used co-rumination to predict the well-being and social distress latent variables, while controlling for the predictors. The model is shown in Fig 1, which depicts the standardized coefficients. It was an acceptable fit to the data, although the RMSEA was slightly larger than recommended for good model fit, χ^2^(13) = 55.13, χ^2^/df ratio = 4.24, CFI = .98, RMSEA = .085, SRMR = .024. Perspective-taking, *β* = .20, *p* < .001, extraversion, *β* = .12, *p* = .014, and younger age, *β* = −.18, *p* = < .001, all predicted co-rumination. In turn, co-rumination predicted less relationship distress, *β* = −.14, *p* = .002, but more anxiety, *β* = .20, *p* < .001, and was unrelated to well-being, *β* = .08, *p* = .19.

Tests of indirect effects showed that co-rumination mediated the relationship between age and social distress, *IE* = .024, *p* = .011, such that older age was associated with less co-rumination, which, in turn, was associated with less social distress. Co-rumination also mediated the relationship between perspective-taking and social distress, *IE* = −.027, *p* = .022, such that perspective-taking was associated with greater co-rumination, which in turn was associated with less social distress. Co-rumination mediated the relationship between age and anxiety, *IE* = −.034, *p* = .002, such that older age was associated with less co-rumination, which in turn was associated with greater anxiety. Co-rumination mediated the relationship between perspective-taking and anxiety as well, *IE* = .040, *p* = .005, such that greater perspective-taking was associated with greater co-rumination, which in turn was correlated with greater anxiety. Co-rumination also mediated the relationship between extraversion and anxiety, *IE* = .022, *p* = .028, such that extraversion was positively associated with co-rumination, which in turn was associated with greater anxiety.

## Discussion

Being social animals, people are sometimes drawn to repetitively process their problems with others. Most prior research on this phenomenon has been conducted in adolescent and young adult samples, with a meta-analysis of 38 co-rumination studies reporting an age range of 12–22 years [12]. In these studies, co-rumination is common and generally has both social benefits and emotional costs [5,26]. To extend our understanding across the lifespan, we examined a tentative model of the correlates of co-rumination, including demographic characteristics, personality and social cognition traits, and measures of social distress, anxiety, and well-being in a large, online representative sample of adults in the UK.

With respect to demographics, the lack of an association between gender and co-rumination was inconsistent with both the individual rumination literature [40] and the adolescent co-rumination literature, which has consistently found that females score more highly on self-reported co-rumination [5,12]. One explanation for this discrepancy is that the greater tendency to co-ruminate in females is largely confined to adolescence, reflecting sociocultural norms during that developmental stage. The highly central role of friendships for adolescents is especially pronounced for girls, who report having more and higher quality friendships than boys [41]. Our study raises the possibility that this tendency to co-ruminate “evens out” across the lifespan to become equally common in adult men and women.

Consistent with previous research, co-rumination was more common in younger people than older people. This finding suggests that this style of shared, repetitive problem-solving may have a more prominent role in young adulthood as identity is evolving toward a coherent self-concept [42]. During this period, young people spend much of their time with peers, who tend to have a more influential role in making decisions and solving problems. This fairly normative co-rumination process may become more indicative of negative emotionality as people age, with a recent meta-analysis finding that the positive correlation between co-rumination and depressive symptoms was stronger in adults [43]. Longitudinal research is needed to better understand how co-rumination and its impacts change across the lifespan.

Turning to individual differences, co-rumination was associated with traits and abilities that are generally associated with adaptive functioning, such as extraversion, agreeableness, and better social cognition, and negatively correlated with rejection sensitivity, a trait associated with worse psychological health [44,45]. Given the interpersonal nature of all of these constructs, the pattern suggests that co-rumination reflects sociability, social connectivity and a resultant tendency to process problems with others. The link between increased perspective taking and the tendency to dwell on problems and share negative experiences with others is consistent with a recent study of adolescents [46], which found that social perspective-taking was associated with both positive friendship quality and co-rumination. The link between better social cognition and co-rumination differs from individual rumination, an inherently more self-focused process, which is associated with decreased awareness of others’ perspectives [15].

The social correlates of co-rumination in the current study were also beneficial, including a positive relationship with well-being and a negative association with loneliness, which is consistent with the adolescent literature [3]. Co-rumination was expected to show a positive association with feelings of relationships well-being, as by definition it involves engaging in intense emotional conversations with others. However, we are unable to rule out the possibility that these benefits associated with co-rumination are an artefact of one having more social interpersonal interactions overall. Future studies may examine this experimentally, which will allow experimenters to hold constant the overall amount of interpersonal contact and manipulate the style of the interaction.

In this adult community sample, we found partial support for the trade-off hypothesis that has been observed in adolescents such that the positive relationship between co-rumination is offset by internalizing symptoms [26,47]. Previous findings suggest a small to moderate association between co-rumination and internalizing problems [12,43], but suggested that the association between co-rumination and depressive symptoms was weaker in adolescents compared to undergraduates and adults. In the current study, co-rumination was not associated with depression, and in fact was positively associated with psychological, physical, and relationships well-being. Nonetheless, we replicated the finding that co-rumination was associated with anxiety, which may represent one emotional cost of the repetitive non-productive focus on problems [12]. Alternatively, more anxious people may engage in more co-rumination to alleviate their anxiety. However, our findings that extraversion and perspective-taking were associated with greater co-rumination suggests that individuals who seek out social contact and have good social-cognitive abilities may be attracted to opportunities to co-ruminate. Yet co-rumination is associated with increased anxiety in these people. Future experimental work is needed; however, to test a potential causal role for co-rumination in increasing anxiety among more socially extroverted individuals. Future studies with clinical samples will allow researchers to draw clinically meaningful conclusions. The findings here provide clues as to possible relationships with anxiety symptoms that could be followed up in clinical studies.

Although our study was cross-sectional and correlational, we evaluated a preliminary model of co-rumination that might be later tested in laboratory research during real-time exchanges. As expected, our social cognition, personality and demographic variables (perspective-taking, extraversion, and younger age) predicted co-rumination, which in turn, predicted less relationship distress. This supports an interpersonally-oriented, and predominantly beneficial, model of co-rumination in which younger, more socially skilled individuals are more likely to process their problems with others, which leads to closer, healthier relationships. However, the results regarding the Big 5 should be interpreted cautiously as internal consistency reliability estimates were low for agreeableness and openness. Furthermore, in our SEM model, the CFI and SRMR values indicated excellent fit, but the RMSEA was slightly larger than that typical of excellent-fitting models. In addition, because our sample is representative only of UK residents, and owing to potential self-selection bias, caution should be used when extrapolating the findings.

Despite these limitations, we hope that the research presented here will serve as a springboard for future investigations of co-rumination. For instance, our co-rumination measure allowed individuals to specify any confidant that they co-ruminated with. This could have been a friend, co-worker, relative, romantic partner, or any other close person. It remains to be seen whether relationship type moderates the associations between co-rumination and well-being. For example, we expect that two co-workers co-ruminating about potential layoffs might elicit more anxiety than co-rumination between one anxious worker and their supportive romantic partner. Moreover, technology provides ever-increasing opportunities for co-rumination outside of traditional interpersonal dyads and irrespective of geographical proximity. In particular, social media platforms provide unprecedented opportunities for analyzing our problems with others; all of us have the ability to co-ruminate with another person on the other side of the world, immediately and in real-time, at our finger tips. Investigations of cyber co-rumination will therefore be an important direction for future research. For example, whether the relationships we observed in this study would be replicated for co-rumination that occurs in digital contexts is an empirical question for future research.

Future studies could also address unanswered questions about the interplay of co-rumination and well-being across the lifespan. For example, it may be the case that social relationships play a more central role in the lives of young people relative to older individuals. Perhaps as people age, they have fewer opportunities to co-ruminate – making it more likely that they will engage in individual (i.e., rather than co-) rumination. Future longitudinal research could test this hypothesis. Finally, as is the case for individual rumination, the effect of co-rumination may depend on context and processing style (e.g., [2]). Experimental studies are needed to test whether there are forms of co-rumination that are beneficial (e.g., concrete) and others that are less so (e.g., abstract). Other approaches, such as ecological momentary analysis (EMA) would enable real-time assessment and allow a clearer evaluation of co-rumination’s short-term emotional and social consequences.

In summary, this study provides initial evidence that in adults, co-rumination may predominantly be associated with adaptive social outcomes, yet at the same time have a positive association with anxiety. Younger adults, and those high in extraversion and perspective-taking are more likely to report higher levels of co-rumination.
